# Prospects of microgreens as budding living functional food: Breeding and biofortification through OMICS and other approaches for nutritional security

**DOI:** 10.3389/fgene.2023.1053810

**Published:** 2023-01-25

**Authors:** Astha Gupta, Tripti Sharma, Surendra Pratap Singh, Archana Bhardwaj, Deepti Srivastava, Rajendra Kumar

**Affiliations:** ^1^ Sharda School of Agricultural Sciences, Sharda University, Greater Noida, India; ^2^ Plant Molecular Biology Laboratory, Department of Botany, Dayanand Anglo-Vedic (PG) College, Chhatrapati Shahu Ji Maharaj University,, Kanpur, India; ^3^ Molecular Biology and Biotechnology Division, CSIR-National Botanical Research Institute, Rana Pratap Marg, Lucknow, India; ^4^ Department of Agriculture, Integral Institute of Agricultural Science and Technology, Integral University, Lucknow, Uttar Pradesh, India; ^5^ Division of Genetics, ICAR-Indian Agricultural Research Institute, New Delhi, India

**Keywords:** biofortification, functional food, microgreen, nutrition, OMICS, shelf life

## Abstract

Nutrient deficiency has resulted in impaired growth and development of the population globally. Microgreens are considered immature greens (required light for photosynthesis and growing medium) and developed from the seeds of vegetables, legumes, herbs, and cereals. These are considered “living superfood/functional food” due to the presence of chlorophyll, beta carotene, lutein, and minerals like magnesium (Mg), Potassium (K), Phosphorus (P), and Calcium (Ca). Microgreens are rich at the nutritional level and contain several phytoactive compounds (carotenoids, phenols, glucosinolates, polysterols) that are helpful for human health on Earth and in space due to their anti-microbial, anti-inflammatory, antioxidant, and anti-carcinogenic properties. Microgreens can be used as plant-based nutritive vegetarian foods that will be fruitful as a nourishing constituent in the food industryfor garnish purposes, complement flavor, texture, and color to salads, soups, flat-breads, pizzas, and sandwiches (substitute to lettuce in tacos, sandwich, burger). Good handling practices may enhance microgreens’stability, storage, and shelf-life under appropriate conditions, including light, temperature, nutrients, humidity, and substrate. Moreover, the substrate may be a nutritive liquid solution (hydroponic system) or solid medium (coco peat, coconut fiber, coir dust and husks, sand, vermicompost, sugarcane filter cake, *etc.*) based on a variety of microgreens. However integrated multiomics approaches alongwith nutriomics and foodomics may be explored and utilized to identify and breed most potential microgreen genotypes, biofortify including increasing the nutritional content (macro-elements:K, Ca and Mg; oligo-elements: Fe and Zn and antioxidant activity) and microgreens related other traits viz., fast growth, good nutritional values, high germination percentage, and appropriate shelf-life through the implementation of integrated approaches includes genomics, transcriptomics, sequencing-based approaches, molecular breeding, machine learning, nanoparticles, and seed priming strategiesetc.

## 1 Introduction

In the past few decades, interest in organic and nutritional vegetables has gained momentum among people. That has increased the demand for sprouts and microgreens. Sprouts are considered germinated seeds that can be harvested before the growth of true leaves and consumed whole with the seed (Di Gioia et al., 2017). On the other hand, microgreens are defined as tender, immature greens that need light for photosynthesis, growing medium (soil or nutrient solution medium), and represent a 7–28 days growth cycle. Microgreens can be developed from the seeds of cereals, vegetables, legumes, and herbs, comprising two completely expanded cotyledon leaves with or without the appearance of a rudimentary pair of first true leaves (Xiao et al., 2012). The history of microgreen production can be traced back to the 1980s when it first seemed on the menu of chefs in San Francisco, California (United States Department of Agriculture, 2014). Following its popularity further, its cultivation began in the southern part of California in the 1990s, such that now microgreens are regarded as “functional foods” or “Superfoods.”

Some cereals (rice, corn, oats, wheat, and barley) and legumes (chickpeas, beans, and lentils) can also be exploited for microgreens cultivation and production due to their nutritional values. Microgreens may be used for sweet and savory dishes as a garnish. They can complement the flavor, texture, and color of salads, soups, flatbreads, pizzas, and sandwiches (alternative to lettuce in tacos/burgers/sandwiches) due to a good proportion of nutritional values and some specific metabolites. People can also supplement microgreens to prepare smoothies, juices, and health drinks. Several value-added products can be synthesized by using fresh and dry microgreens in food science laboratories as one ingredient, for example, cookies, noodles, snacks, and chips are developed. Microgreens products would be an up-and-coming resource for vegetarians as a healthy lifestyle change and generation of employment for the population engaged in agriculture and food processing sectors.

Very few reports are available on the cultivation of legumes microgreens, for example, chickpea micro-greens (Sreenivasan, 2020). The small-seeded legumes promise unmarked resources of potential ingredients for the fortification of traditional or staple foods with bioactive compounds, nutrients, and minerals (Butkutė et al., 2018). Erosion of preserved seed nutrients was observed in chickpea during the process to stimulate seedling growth that improved nutritional value with digestible protein levels ranging from 18.96% to 28.69% in 6-day-old sprouts identifying the genotypes BG-1092, ICC-11378, JG-74 as potential resources for accumulating proteins and other nutritional components at the seedling stage (Kumar et al., 2022a). Therefore, the cultivation of microgreens can be easily practiced, and cheaper sources of budding superfoods in terms of nutrition and antioxidant properties with minimum food wastage (only root) during consumption may be obtained. Thus, in the present report, we have discussed the prospects of nutraceutical aspects, health benefits, growth, and cultivation practices for microgreen production and mentioned related strategies for the first time to overcome the limitations through the utilization/integration of various OMICS approaches.

## 2 Nutraceutical and health benefits of microgreens: The superfoods/functional foods

Nutrients deficiency may cause serious diseases and health related issues and appropriate proportions of micronutrients, macronutrients, flavonoids and polyphenols regulate immunity and prevent from several health threats viz; osteoporosis, pharmacotherapy, COVID-19 etc (Batiha et al., 2022; Martiniakova et al., 2022; Bansal et al., 2022a; Bansal et al., 2022b). Microgreens are partially mature greens that elicit their intense flavour, aroma, texture, and nutrient properties with sensory attributes and acceptance are discussed in this review. Desirable sensory qualities and intense flavours of these superfoods have gained acclamation to be consumed as salads, garnishes, *etc.* The presence of minerals, vitamins, and their precursors - ascorbic acid and several other bioactive compounds tocopherols, carotenoids, betaine, phenols, glucosinolates, phytosterolsetc in microgreens add to their health and nutrition-related functional aspects (Xiao et al., 2012; Kyriacou et al., 2019a)as presented in Table 1 and Table 2. The sensory qualities of microgreens are influenced by their chemical composition (Xiao et al., 2015). This study highlights the correlation between the concentration of total phenols and with overall acceptability of sensory attributes and acceptance in terms of sweetness, sourness, bitterness, and astringency. The sensory and nutritional qualities of microgreens also vary with the growing methods. The sensory attributes and nutritional content of microgreens grown hydroponically and in soil procured from a commercial and local farm were compared (Tan et al., 2020). Nutritionally microgreens grown by either method were comparable. However, a significant difference in vitamin C content was reported. Variations in both micro-minerals (iron, zinc, copper, and manganese) and macro-mineral content (calcium, phosphorus, sodium, magnesium, chloride, potassium, and sulfur),phytochemical profile, and antioxidant capacities have been found to vary with genotype rather than growth stage as studied in microgreens of four Brassicaceae genotypes-Komatsuna, Mizuna, Pak Choi and Mibuna (Kyriacou et al., 2021a). In addition, delaying harvest from the arrival of the first to second true leaf does not seem relevant for improving bioactive compounds in microgreens (Kyriacou et al., 2021a). Regulated feeding of nutrient solution (NS) to microgreens and fertigation treatment can also influence the composition of phytochemicals and antioxidant activity apart from growth and yield (Petropoulos et al., 2021). Moreover, spinach microgreens were assessed for the effect of nutrient deprivation (0, 5, 10, and 20 days) and fertigation treatment before harvesting (Petropoulos et al., 2021). This study reported that NS feeding for a longer duration of 20 days resulted in enhanced fresh yield and content of photosynthetic pigments, including chlorophyll, beta carotene, and lutein. In contrast, the concentration of minerals like Calcium (Ca), Potassium(K), magnesium (Mg), and Phosphorus (P) were found to be lowest after 20 days in contrast to being maximum in control and 5 days of NS feeding (Petropoulos et al., 2021). Feeding the spinach microgreens for 10 days with NS yielded the best combination of yield, minerals (high), and nitrate (low) while maintaining the concentrations of bioactive compounds (Petropoulos et al., 2021). Simultaneous to the accumulation of secondary metabolites, microgreens are also known to accumulate anti-nutritive compounds like nitrate. Therefore, nutrient deprivation before harvest (DBH) was employed to reduce nitrate levels by substituting NS with osmotic water for 6 and 12 days in a garden rocket, lettuce, and mustard microgreens grown on a peat-based substrate (Kyriacou et al., 2021b). Nutrient deprivation proved a good strategy for lowering nitrate content with effective treatment duration varying from species to species. Even nitrate hyper-accumulating species like garden rockets showed an abrupt decline in nitrate concentration (Kyriacou et al., 2021b). Further, abundant secondary metabolites like flavanol glycosides, quercetin, and kaempferol glycosides were detected in Brassicaceae*,* and caffeoyl quinic acid in lettuce microgreens. However, total phenols increased in lettuce, reduced in the garden rocket, and unaffected in mustard microgreens in response to nutrient deprivation (Kyriacou et al., 2021b).

**TABLE 1:** Assessment of microgreens studies in different plants.

S.No.	Crop species	Objective of study	Key findings	Conclusion/Recommendation	References
**Vegetables**					
1	Cabbage, Kale, Argula and Mustard	To study yield and appearance quality in response to variation in blue light	Fresh and dry weight remained unaffected, however hypocotyl length and cotyledon area decreased	Blue light 15% and 5% were best for cabbage other three microgreens respectively	Ying et al. (2020a)
2	Kale (*Brassica napus* L. ‘Red Russian’), mustard (*Brassica juncea* L. ‘Ruby Streaks’), cabbage (*Brassica oleracea* L.), and arugula (*Eruca sativa* L.).	To study effect of Single Source (SS) LED on growth, yield and quality. Further, to develop mathematical models to understand these relationships	Increased fresh and dry weight with increase in light intensity while hypocotyl length and hue angle decreased linearly. Phenotypic plasticity exhibited by arugula and mustard were greater compared to kale and cabbage	Optimum Sole Source-LED light intensity for these four microgreens depending on genotype, production system and goal	Jones-Baumgardt et al. (2019)
3	Arugula, cabbage, mustard and kale	To study the effects of photoperiod shortening on elongation growth	Blue light promoted elongation was evident from length of petiole and rate of stem extension	Blue light promotes elongation growth for 16–24 h photoperiod and is beneficial for indoor production methods	Kong et al. (2019)
4	Mustard and arugula	To study the effect of treatment of Blue light and combined effect of Blue and Far-Red light during night on yield, plant quality and elongation	Under Blue light plant height increased by 34% and 18% in mustard and arugula respectively. Combination of B and FR light also gave similar results without compromising with yield and quality of microgreens under either treatment conditions	Treatment with blue light alone at night can promote elongation in microgreens while maintaining their yield and quality	Ying et al. (2020b)
5	Broccoli	To study effect of application of CaCl_2_ pre-harvest and UV-B post-harvest on levels of Glucosinolates (GLS) and glucoerucin (GLE) i.e. storage quality of microgreens	Content of total aliphatic glucosinolates in microgreens was four times as compared to mature counterparts. Treatment with 10 mM CaCl_2_ and UV-B also enhanced GLS levels	Spraying microgreens with CaCl_2_ prior to harvest not only enhances levels of GLS it also improves visual appearance and storage/shelf life, alongwith UV-B exposure post- harvest	Lu et al. (2018)
**Cereals**					
1	*Triticum aestivum* (wheat) and *Hordeum vulgare* (barley)	Profiling content of chlorophyll and carotenoid during 7 and 16 days on dry basis and evaluate pigment accumulation rate	Content of chlorophylls and carotenoids had strong correlation with number of growth days and progressively increased up to day 16. Accumulation of pigments was maximum between day 7–10 in wheat and day 10–13 in barley	Cereal microgreens can be considered for *in vivo* studies for potential use in nutraceutical and pharmaceutical industry	Niroula et al. (2019)
**Herbs—Medicinal/Culinary**					
1	*Daucas carota* L. var. *New Kuroda, Foeniculum vulgare* Mill., *Trigonella foenum –graecum* L., *Ocimum basilicum* L., *Alium cepa* L. var. *Light Red Gavran, Hibiscus sabdariffa* L. (white var.), *Raphanus sativus* L. var. *Imp. Chetki, Spinacia oleraceae* L. var. *All Green, Helianthus annus* L., *Brassica juncea* L	To study the bioactive phytochemicals (Overall phytochemical composite index -OPCI)and overall antioxidant activity measured as Antioxidant potential composite index APCI using appropriate parameters), of ten culinary microgreens. Further, the phytochemicals contributing in antioxidant potential were also identified	Antioxidant potential and Phytochemical profile was reported highest in *Foeniculum vulgare* Mill. And *Hibiscus sabdariffa* L. microgreens. Total phenols and total flavonoids contributed maximum to OPCI, APCI and radical scavenging activity	Wholesome nutritional status of ten culinary microgreens was reported	Ghoora and Srividya, (2020)
**Legumes**					
1	Lentil and Mung bean	Analysis of diversity in phytochemical profile, antioxidant capacity and content of micro as well as macro nutrients in 20 genotypes of lentil and mung bean grown in plain and high altitude regions	L830 and MH810 genotypes of lentil and mung bean respectively, were identified as superior based on antioxidant activity, total flavonoids, ascorbic acid, carotenoids, and phenol content	Nutritional profiles of same genotypes showed variation when grown in two different altitude regions of Delhi and Leh –Ladakh	Mishra et al. (2021)
2	Black Gram, Chickpea and Mung bean	To analyse and compare the nutritional profile of these legumes cultivated in water, soil and cocopeat supplemented with nutrient solution	Mineral content, phenol proximate and amino acid composition, antioxidant activity showed variation in different species and substrate used for cultivation	Nutritional qualities of the crops varied with substrate used and crop species	Kaur et al. (2022)
3	*Trifolium pratense*	To determine the nutritional potential, phytochemical and mineral profile of forage legumes by analysing the seeds, sprouts and microgreens	All species exhibited high nutritional potential, phytochemical and mineral values. Mineral and protein levels were high in all three forms studied. Increase in quantity of protein and phytochemicals was observed from seed to microgreen stage, however trends were opposite for total carbohydrates	Small seeded legumes, (especially as microgreens) have the potential of being used for fortification to enhance nutrients as well as bioactive compounds in staple food	Butkutė *et al.* (2018)
	*T. medium, Medicago sativa, M. lupulina, Onobrychis viciifolia, Astragalus glycyphyllos* and *A. cicer*				
4	*Vigna radiata* L. (Mungbean), *Lens culinaris* subsp. *culinaris* (lentil), and *Brassica juncea* L. (Indian mustard)	To optimize and evaluate yield, shelf-life, sensory parameters and microbial load in microgreens of these crop species	Optimum seed density (to obtain maximum yield), time of harvest was reported for three species. High correlation between seed size and yield for both legume species. No pathogenic bacteria were found in microbial load	Proper cultivation and storage of microgreens can aid in their safe human	Sangwan et al. (2022)
5	Alfalfa, Fenugreek, Lentil, and Daikon Radish	To determine polyamine content in seed, sprouts and microgreens of these crops and find the stage with superior quantity of polyamines. Also to determine the enzymatic capacity of sprouts to degrade unwanted biogenic amines	Polyamines of nutritional importance (spermine, spermidine and agmatine) were in abundance in microgreens. Cadaverine was highest in sprouts of legumes. On the other hand, nutritionally important polyamines were higher in sprouts of daikon radish than their microgreen counterparts	Microgreens are a rich source of nutritionally beneficial polyamines	KraljCigic et al. (2020)

**TABLE 2:** Comparative analysis of nutritional components in microgreeens, mature part and different stages of plants.

Table-2				
Plant	Immature/Microgreen (7 days after seed sowing)	Seedling (15 days after seed sowing)	Mature (adult stage-30 days after seed sowing	References
*Brassica rapa* subsp. Chinensis var. Parachinensis (Choy sum)	**Essential amino acids**- 15.8	**Essential amino acids**- 7.8%	**Essential amino acids**-10.4%	Zou et al. (2021)
	**Total concentration of sugars**- 0.01g/100 g FW	**Total concentration of sugars**-0.2 g/100 g FW	**Total concentration of sugars**- 0.2g/100 g FW	
	**vitamin B9** (folate)	**vitamin B9** (folate)	**vitamin B9** (folate)	
	CHO-folate- 46 ± 5 µg//100 g FW	CHO-folate- 46 ± 4 µg/100 g FW	CHO-folate-31 ± 7 µg/100 g FW	
	Folic Acid- 1.6 ± 0.22 µg//100 g FW	Folic Acid-3.0 ± 1.26 µg//100 g FW	Folic Acid- 2.6 ± 1.57 µg//100 g FW	
	Tetrahydrofolate- 36 ± 6 µg//100 g FW	Tetrahydrofolate- 34 ± 5 µg//100 g FW	Tetrahydrofolate- 33 ± 9 µg//100 g FW	
	5-methyltetrahydrofolate -23 ± 2 µg//100 g FW	5-methyltetrahydrofolate- 32 ± 2 µg//100 g FW	5-methyltetrahydrofolate-20 ± 3 µg//100 g FW	
	**Lipid soluble Vitamin A**	**Lipid soluble Vitamin A**	**Lipid soluble Vitamin A**	
	β-Cryptoxanthin- 97 ± 19 µg//100 g FW	β-Cryptoxanthin- 101 ± 18 µg//100 g FW	β-Cryptoxanthin- 35 ± 48 µg//100 g FW	
	Neoxanthin- 2,105 ± 279 µg//100 g FW	Neoxanthin - 2,909 ± 490 µg//100 g FW	Neoxanthin -2,243 ± 486 µg//100 g FW, Violaxanthin - 2,201 ± 602 µg//100 g FW	
	Violaxanthin- 2040 ± 296 µg//100 g FW	Violaxanthin - 4,336 ± 731 µg//100 g FW		
	**α-Tocopherol & γ-Tocopherol:** no significant change at 3 stages			
	**Vitamin K1**- 377 ± 29 μg/100 g FW	**Vitamin K1**- 433 ± 33 μg/100 g FW	**Vitamin K1**- 363 ± 27 μg/100 g FW	
	**Glucosinolates:** Gluconapoleiferin-517 ± 125 μg/100 g FW, Gluconapin - 5,576 ± 1,431 μg/100 g FW	**Glucosinolates:** Gluconapoleiferin **-** 214 ± 40 μg/100 g FW, Gluconapin - 952 ± 257 μg/100 g FW	**Glucosinolates:** Gluconapoleiferin**-** 85 ± 16	
			μg/100 g FW, Gluconapin - 3,488 ± 181 μg/100 g FW	
	**Minerals** (μg/100 g FW)	**Minerals** (μg/100 g FW)	**Minerals** (μg/100 g FW)	
	Copper (Cu)- 27 ± 4 Iron (Fe) - 504 ± 77, Magnesium (Mg)- 30,411 ± 5,705, Potassium (K)- 411,908 ± 46,579, Zinc (Zn)- 472 ± 80	Copper (Cu)- 21 ± 8, Iron (Fe)- 358 ± 77, Magnesium (Mg)- 22,504 ± 4,685, Potassium (K)- 229,953 ± 40,028, Zinc (Zn)- 412 ± 93	Copper (Cu) - 20 ± 5, Iron (Fe)- 325 ± 48, Magnesium (Mg)- 26,838 ± 5,994, Potassium (K)- 222,630 ± 69,219, Zinc (Zn)- 280 ± 55	
Broccoli microgreens	Copper and selenium were 13.7 fold higher in microgreens as compared to the mature stage	**-**	**-**	Johnson et al. (2021)
Red cabbage microgreens	2.1 fold higher phosphorus, 2.4 fold higher iron, 3.8 fold higher zinc and 9.1 fold higher copper contents were explored in microgreens in comparison to mature stage	-	-	Johnson et al. (2021); Podse dek et al. (2006)
	Vitamin E (0.06 mg/100 g FW) was forty times higher in comparison to the mature red cabbage			
Red beet microgreens	10.2 fold higher selenium, 3.1 fold and 2. 5 fold higher chromium	-	-	Johnson et al. (2021)
Red amaranth microgreens	2.2 times higher concentration of copper than the fully developed plants	-	-	Johnson et al. (2021)
Pea microgreens	12.2 and 16.8 times higher contents of molybdenum and selenium were recorded in comparison to 38 days old pea plants	-	-	Johnson et al. (2021)
Golden pea	Quantity of α-tocopherol and γ-tocopherol was significantly higher (4.9 mg/100 g FW and 3.0 mg/100 g FW respectively) than mature spinach leaves (α-tocopherol- 2.0 mg/100 g FW; γ-tocopherol -0.2 mg/100 g FW)	-	-	Xiao et al. (2012)
Cilantro microgreens	5 times and 2.8 times more violaxanthin content was estimated as observed in the mature leaves of cilantro (1.4 mg/100 g FW) and spinach leaves (2.7 mg/100 g FW) respectively	-	-	Bunea et al. (2008); Kobori and Amaya, (2008)

The presence of bioactive compounds renders microgreens as a health beneficiary, antioxidant,anti-microbial, anti-inflammatory, and anti-carcinogenic. The health-related properties of bioactive compounds in food and herbs depend not only on their content and the amount consumed but also on their bioavailability (de la Fuente et al., 2019). Quantity and bio-accessibility of bioactive antioxidant compounds (total anthocyanins, total soluble polyphenols, ascorbic acid, total isothiocyanates), antioxidant capacity (Trolox Equivalent Antioxidant Capacity, and Oxygen Radical Absorbance capacity), macro-elements (K, Ca and Mg) and oligo elements (Fe and Zn) have been evaluated in four hydroponic Brassicaceae microgreens-broccoli, radish, kale and mustard (de la Fuente et al., 2019). The optimum amount of nutrients (macro and oligo-elements) is required for proper growth metabolism, and in contrast, deficiency may lead to life-threatening diseases in extreme circumstances. The essential nutrients are widely distributed in foods, and most people can obtain sufficient amounts by consuming a varied diet. Moreover,macro-elements (K, Ca, and Mg) and oligo-elements (Fe,Se, Cu, and Zn) remarkably stimulate the function of the immune system and are helpful in cardiopulmonary bypass (Al-Bader et al., 1998). Microgreens of soybean, green pea, garden rocket, radish, and red Rambo radish were cultivated under fluorescent and LED light conditions. The variation in anti-proliferative/pro-oxidant efficiencies of these microgreens was studied using Ewing sarcoma lines RD-ES and A673 (Truzzi et al., 2021). It was observed that all microgreen extracts could reduce cell proliferation in 2-dimensional cell cultures, while extracts from pea microgreens grown under LED light showed anti-proliferative and pro-apoptotic activity on 3-dimensional A673 and RD-ES spheroids without showing cytotoxicity on healthy L929 fibroblasts (Truzzi et al., 2021). LED and fluorescent light illuminated Red Rambo radish also exhibited anti-tumor effects on RD-ES spheroids. Further, the effects of UV-A, B, and C on inducing polyphenol content and anti-tumor activities of UV-illuminated microgreens can be an area of exploration in the future. In a study, the effect of salinity in combination with different wavelengths of light in *Brassica carinata* extracts from microgreens grown under different treatments of salinity and light were checked for their ability to stimulate antioxidant enzymes, including catalase (CAT), superoxide dismutase (SOD) and expressions of Nrf2 (nuclear transcription factor-erythroid 2 related factor) and HO-1 proteins (heme-oxygenase -1)on human colorectal carcinoma cells-HCT116 (Maina et al., 2021). Activation of antioxidant enzymes (SOD and CAT) and stimulation of HO-1 and Nrf2 make them preferable for the prevention and treatment of oxidative stress and inflammatory disorders (Maina et al., 2021). Microgreens can be a powerful source of nutrients owing to higher concentrations of several phytochemicals than their matured counterparts. These can be used as supplements to overcome deficiencies of several nutrients. Being a rich source of bioactive compounds like carotenoids, phenols, glucosinolates, polysterols, and many others, microgreens possess health benefits due to anti-microbial, antioxidant, anti-inflammatory, and anti-carcinogenic properties (Choe et al., 2018; Le et al., 2020).

Most of the microgreens viz., broccoli, red beet, red amaranth, red cabbage and pea microgreens exhibit high proportion of minerals and nutritionally rich components (copper, selenium, phosphorus, iron, zinc, molybdenum and chromium) as compared to mature and other counterparts (Johnson et al., 2021) as presented in Table 2 and are being narrated as given further.

## 3 Comparison for nutrients and phytochemicals in microgreens and different stages of crop plants

The comparative nutrient profiling was explored in *Brassica rapa* subsp. Chinensis var. Parachinensis (Choy sum) at three different growth stages *viz.* Microgreen, seedling and adult/mature stages (7, 15 and 30 days after seed sowing) respectively (Zou et al., 2021). The content of essential amino acid was high at microgreen stage (15.8%) as compared to seedling (7.8%) and mature (10.4%) stages. Moreover, the contents of metabolites and minerals were estimated along with their changes at maturity in six crops microgreens belonging to three distinct families Brassicaceae (*Eruca sativa* L.) Cav. - Arugula, *Brassica oleracea* L. Italica Group-Broccoli and *Brassica oleracea* L. Capitata Group - Red cabbage), Fabaceae (*Pisum sativum* - Pea) and Amaranthaceae—(*Amaranthus tricolor* L. -Red amaranth and *Beta vulgaris* L. Crassa Group- Red beet) (Johnson et al., 2021). Moreover, metabolites showed significant difference (*p* < 0.05) and were ≥2- times greater in microgreens as compared to their mature counterparts (Johnson et al., 2021). There are 95 such metabolites in broccoli, 110 in red cabbage, 87 in arugula, 80 in red beet, 93 in pea and 101 in red amaranth microgreens, respectively. These metabolites were majorly classified as peptides, saccharides, nucleotides, amines, phenolics, lipids, organo-sulfurs, alkaloids and vitamins with prevalence of lipids and phenolics (Johnson et al., 2021). In another study, good quantities of α-tocopherol and γ-tocopherol were estimated in golden pea tendrils as 4.9 mg/100 g FW and 3.0 mg/100 g FW respectively (Xiao et al., 2012). Interestingly, cilantro microgreens and red cabbage microgreens have been reported to have more violaxanthin and vitamin E contents as compared to their mature counterparts, respectively (Podse dek et al., 2006; Bunea et al., 2008; Kobori and Amaya, 2008).

Further, now it is a proven fact that the red cabbage microgreen regulates the levels of lipids, cholesterols in human blood vessels and protects against cardiovascular diseases (Huang et al., 2016). The mustard and coriander microgreens have been reported to be very rich sources for their antioxidant, antimicrobial, anti-cancerous, anti-obesity, anti-inflammatory and antidiabetic activities (de la Fuente et al., 2020; Le et al., 2020; Saengha et al., 2021; Truzzi al., 2021; Dhakshayani and Alias 2022). Hence, appropriate daily consumption of microgreens would be a potential protective nutritional strategy to manage health, nutrition and chronic degenerative diseases (de la Fuente et al., 2020).

## 4 Microgreens: A novel, live, super functional food

Microgreens’ demand and preference are attributed to their aroma, tender texture, vivid colour, flavour, sensory attributes, and quick production or cultivation. Several microgreen species have peculiar colours; for example, microgreens of broccoli, spinach, and celery have been reported to be green in colour, microgreens of radish, red basil, and red cabbage are crimson, while multicolour microgreens have been developed in mustard and beet (Di Gioia et al., 2015). Some of the previous studies related to microgreens have been summarized in Table 1. Similarly, microgreens are also known to possess a distinct flavour viz; spinach and rapini taste-neutral, arugula, radish, and watercress spicy and *Cucurbitaceae* microgreens as bitter have been reported (Di Gioia et al., 2017).

Most of the species and varieties used in current microgreen production come from the Brassicaceae and Amaranthaceae families (Xiao et al., 2015; Kyriacou et al., 2016). In the Amaranthaceae family, some of the more popular species, subspecies, and varieties include beet, chard, and amaranth; in the *Brassicaceae* family, radish, broccoli, kale, cabbage, tatsoi, pakchoi, mizuna, arugula, and mustard. Microgreens of grain crops such as buckwheat, wheat, and rye have also been grown. Various medicinal and culinary herbs have also been used for microgreen production, including borage (or starflower), parsley, basil, and fenugreek, among many others (Verlinden, 2020). Microgreens’ taste can fluctuate significantly based on the variety. The widely used varieties have been explored for microgreens production from seeds of diverse plant families as mentioned further;Brassicaceae (Cauliflower, broccoli, cabbage, watercress, radish, and arugula), Apiaceae (Dill, carrot, fennel, and celery), Asteraceae (Lettuce, endive, chicory, and radicchio), Amaranthaceae (Amaranth, quinoa swiss chard, beet, and spinach), Amaryllidaceae (Garlic, onion, and leek) and Cucurbitaceae (Melon, cucumber, and squash).

Increasing interest in the production and consumption of microgreens is also due to their high nutritional content, high yield, rapid production, aroma, and other qualities. Their high nutritional qualities are mainly due to the presence of phytochemicals and other bioactive compounds, along with their antioxidant capacities. They are considered highly nutritious food because of the presence of nutrients that include proteins, minerals, vitamins, carotenoids, phenols, and glucosinolates (Ebert, 2013; Di Gioia et al., 2017). The concentrations of bioactive compounds found in microgreens and even sprouts are reported to be much higher than their mature counterparts (Kyriacou et al., 2016). For example, Broccoli microgreens grown hydroponically and in compost were found to have more nutrient content (Mn, Cu, P, K, Na, Mg, and Fe) than mature broccoli vegetables (Weber, 2017). Contrary to this, hydroponically grown fenugreek (*Trigonella foenum-graecum* L.), broccoli, and garden rocket (*Eruca vesicaria subsp. Sativa*) microgreens were reported to have lower mineral contents than their mature plants to be eaten as vegetables. Among the three, only fenugreek microgreens efficiently uptake iron in caco-2 cells (Khoja et al., 2020). Chickpea microgreens also contains a good amount of zinc, calcium, iron, antioxidants, vitamins, carbohydrates, fiber,fat, and high protein content. Moreover, enriched nutrient contents containing plants may produce microgreens with high biomass in a limited time in a cost-effective manner that would improve the cultivation and yield of microgreens.

## 5 Growth and cultivation practices for the production of microgreens

The basic requirements for microgreen cultivation are the availability of substances and the effect of light as narrated below**.**

### 5.1 Availability of substrate for cultivation of microgreens

Various substrates have been used to grow microgreens, and their influence on yield and nutritional quality has been studied. In a study, three different substrates - vermiculite, cotton, and jute fiber were used to grow microgreens of green basil—*Ocimum basilicum* L., Red basil—*Ocimum basilicum* var. Purpurecsens and garden rocket *Eruca sativa* Mill in a Micro Experimental Growing System (MEG) fitted with LED lamps for light supply. In addition to that, several other substrates are also available to use further as primary medium or in combinations, for example, coco peat, coconut fiber, coconut coir dust, coconut husks, sand, jute fiber, vermicompost, sugarcane filter cake, peat and white sphagnum peat substrates presented in Table 3. A high yield of 2–3 kg/m2 was obtained. The three microgreens varied in nutritional quality, with red basil accounting for high antioxidant compounds on vermiculite and jute fiber media. At the same time, the qualitative parameters were found to be species-dependent (Bulgari et al., 2021).

**TABLE 3:** Different growth media/substrate used in different plants for microgreens cultivation.

Crop/Plant	Substrate used	Remarks/Findings	Reference
*Ocimum basilicum* L.- Basil, *Eruca vesicaria* (L.) Cav. subsp. *Sativa* (Mill.) Thell.- Rocket	Hydroponics (Soil-less medium)	High concentrations of some minerals	Bulgari et al. (2017)
*Eruca sativa* Mill. - Rocket, *Ocimum basilicum* L. - Green Basil, *Ocimum basilicum* var. Purpurecsens - Red basil	Vermiculite, coconut fiber, jute	Substrate significantly regulates nitrate concentration, yield and dry matter percentage	Bulgari et al. (2021)
Hairy basil (*Ocimum basilicum* L.f. var. *citratum* Back),	Sand, vermicompost, coconut coir dust, sugarcane filter cake, peat	Local organic biomaterials were identified as suitable substitutes to costly peat-based media for cultivating microgreens.	Muchjajib et al. (2015)
Sweet basil (*Ocimum basilicum* Linn.),			
Holy basil (*Ocimum sanctum* Linn.),			
Huanmoo (*Dregea volubilis* Stapf),			
Sano (*Sesbania javanica* Mig.),			
Vine spinach (*Basella alba* Linn.),			
Rat- tailed radish (*Raphanus sativus* var. caudatus Linn),			
Leaf mustard (*Brassica juncea* Czern. & Coss.),			
Kangkong (*Ipomoea aquatica* Forsk.)			
Krathin (*Leucaena leucocephala* de Wit.),			
Red radish (*Raphanussativus*) var “Sango”	White sphagnum peat substrate, Coco coir dust	Microgreens grown on these substrates had permissible levels of nitrate content and microbial growth.	Thuong and Minh (2020)

### 5.2 Effect of light on nutritional quality and growth of microgreens

Essential growth factors like light (wavelength, intensity, and photoperiod) also influence microgreens’ biosynthesis and accumulation of phytochemicals (Delian et al., 2015). Recently, many studies have been carried out on the effect of artificial light sources like halogen lamps, high-pressure sodium lamps, fluorescent lamps, and LED lights on the growth, yield, nutrient quality, and phytochemical content of microgreens. Many studies have focused on the benefit of illumination with LED light on plant growth, quality, and accumulation of phytonutrients. Some of these studies have been tabulated (Table 4). The effect of different wavelengths of light in combination with salinity was assessed on quality in terms of antioxidant capacity, the content of phenolics and glucosinolates, as well as yield of microgreens of *Brassica carinata* (Maina et al., 2021). Stable cultivation of microgreens was achieved under fluorescent and blue plus red (B1R1) light conditions, which resulted in a high accumulation of biomass and glucobrassicin. Under saline conditions, blue LEDs and fluorescent light promoted antioxidant activity together with the accumulation of phenols, sinigrin and glucosinolate (Maina et al., 2021).

**TABLE 4:** Influence of light on growth and phytochemical quality of microgreens.

S.No	Microgreen	Light treatment	Trait/Aspect studied	Key findings	References
Vegetables					
1	*Brassica juncea* ‘Red Lace’ (Mustard) and Brassica napus‘Red Russian’ (kale)	Blue:red light (LED)	Mineral nutrient content	Increase in blue light decreased elongation and enhanced accumulation of micro as well as macro nutrients	Brazaitytė et al. (2021)
2	Broccoli microgreens	Red:blue:green (1:1:1) LEDs	Growth and phytochemical content	Increased fresh weight, dry weight and moisture content, further, elevated chlorophyll and reduced carotenoid content with increasing light intensity. Contents of some other phytochemicals like vitamin C, soluble proteins and sugar, flavonoid, free amino acid, and glucosinolates except progoitrin also increased	Gao et al. (2021)
3	*Amaranthus tricolor* L. and *Brassica rapa* L. subsp. *oleifera*	White LED, Blue LED and Red LED	Yield and nutritional quality	Blue light is most effective in promoting growth and nutritional qualities. Red light had pronounced effects on accumulation of fresh biomass as well as growth of hypocotyl	Toscano et al. (2021)
4	Chinese Kale	Red, white, Blue LED and sunlight (control)	Growth and antioxidant system	Low intensity red light increased fresh weight ang hypocotyl growth. White LED promoted accumulation of phenolic compounds, glucosinolates and ascorbic acid	Tantharapornrerk et al. (2021)
5	*Eruca sativa* L.- Arugula, *Brassica oleracea* L. var. Capitata f. rubra - red cabbage, Brassica napus L. subsp. napus var. Pabularia - ‘Red Russian’ kale, Brassica juncea L. - ‘Mizuna’ mustard	LEDs supplying blue (5%–30%) and red light (70%–95%)	Phytochemical profiles	20% blue light enhanced ascorbate levels (both reduced and total) in arugula, mustard as well as kale microgreens. 30% blue light stimulated accumulation of phenols in Kale and mustard. Total anthocyanin content showed proportional increase with the % of blue light supplied up to 30 percent in all microgreens, except mustard	Ying et al. (2021)
6	Leafy vegetable amaranth and Red amaranth	LEDs	Growth and nutritional value	Red + blue in ratio 70R:30B (PPFD-280 μmol/m^2^/s^1^; Photoperiod- 16 h) improved fresh yield, vitamin C, content of photosynthetic pigments (carotenoids and chlorophylls), anthocyanins, and levels in both red amaranth and leafy vegetable amaranth microgreens. Further, total antioxidant capacity was also increased	Meas et al. (2020)
7	Amaranth, cress (edible herb), mizuna, purslane	Red, Blue, Blue-Red	Differences in productivity levels, polyphenolic and antioxidant profiles together with content of mineral–carotenoid	Higher nitrate accumulation, Increased concentrations of Na and K, while decreased calcium and magnesium concentration. Enhanced lipophilic antioxidant activity, β-carotene and lutein. Decreased polyphenolic content	Kyriacou et al. (2019b)
8	*Brassica oleracea* var. *gongylodes* (kohlrabi), *Brassica rapa* var. Japonica (mizuna) and *Brassica oleracea* (broccoli)	1^0^ light spectrum comprising of red light (638 and 665 nm), far-red light (731 nm) and blue light (447 nm), or supplemented by yellow (595 nm), green light (520 nm), or orange (622 nm), LED source	Nutrient levels	Metabolic changes resulted in increase in essential nutrients like Iron, Magnesium, Calcium, beta carotene, soluble carbohydrates, ascorbic acid	Samuolienė et al. (2019)
	*Brassica oleracea* var. gongylodes—Kohlrabi, *Brassica rapa* var. *Japonica*—Mizuna and *Brassica oleracea* - Broccoli				
9	Radish	White, Blue, UV-A and dark; light conditions combined with Hydrogen rich water (HRW)	Anthocyanin accumulation	Blue light and UV-A combined with HRW resulted in higher phenolic content. Increased content of anthocyanin compounds	Zhang et al. (2019)
10	Mustard, Beet and Parsley	Blue light treatment	Carotenoid and tocopherol content	Quantity of chlorophylls, carotenoids, alpha-carotenes and beta-carotenes, zeaxanthin, violaxanthin, and lutein, increased 1.2 to 4.3 folds	Samuolienė et al. (2017)
11	*Brassica oleracea* var. *gongylodes*—Kohlrabi, *Brassica juncea* ‘Garnet Giant´- mustard, and *Brassica rapa* var. *Japonica* - mizuna	SS- LEDs; % Ratios as follows: Red_87_:Blue_13_, Red_84_:Far Red_7_:Blue_9_,orRed_74_:Green_18_:Blue_8_	Phytochemical synthesis	Total carotenoids in mizuna and mustard microgreens lowered, as light intensities increased. Higher values of total integrated chlorophyll were observed in kohlrabi at Red_87_:Blue_13_compared to mustard microgreens at Red_84_:Far Red_7_:Blue_9_ and Red_74_:Green_18_:Blue_8._Total concentration of anthocyanins also increased as light intensity increased	Craver et al. (2017)
12	*Brassica rapa* var. *Chinensis—*Red pakchoi, *Brassica juncea* L.—Mustard and *Brassica rapa* var. *rosularis*—Tatsoi	Pulsed LED	Phytochemical levels	Total phenolic content decreased in response to pulsed LED while total anthocyanin content increased	Vaštakaitė et al. (2017)
**Herbs—Medicinal/Culinary**					
1	Chia (*Salvia hispanica* L.) (Dark grown)	Constant light (100 µmol photons/m^2^/s) for 24h and 48 h	Antioxidant activity and metabolic profile	Significant increase in antioxidant activity, chlorophyll and carotenoid synthesis, total soluble phenols and ascorbic acid content	Mlinarić et al. (2020)
2	*Ocimumbasilicum* L. (Acyanic and cyanic basil)	Light Emitting Diodes - Red andBlue	Microgreen morphometric parametres and bioactive compounds	Blue light illumination affected growth parameters resulting in increased cotyledon surface area, fresh weight, anthocyanin concentration and chlorophyll levels. Red light triggered synthesis of phenols and capacity to scavenge free radicals in green cultivar while in red cultivar blue light was found effective for same	Lobiuc et al. (2017)
**Legumes**					
1	Soybean	LED light spectra	Growth, antioxidant capacity, phenolic compounds profile	Decreased seedling heightas well as yield while increase in phenols. UV-A and Blue light significantly increased antioxidant capacity, total phenols and total flavonoid content	Zhang et al. (2019)

## 6 Limitations of microgreen production

The consumption and storage of microgreens pose several limitations. Poor shelf life and postharvest management of microgreens are major challenges for the concerning researchers. It is important to know if a correlation exists between produced spoilage and contamination by the human pathogen. Studies have reported seed-to-harvest pathogen infection in several microgreens like basil, lettuce, parsley, melons, and spinach (Alegbeleye et al., 2018). Growing conditions that promote microorganism growth or transfer, processing practices that expose the commodity to contaminants from animals or humans, and physiological characteristics of the plant that allow contact and binding with microorganisms, all of these factors put crops at risk (Maluin et al., 2021). As microgreens are grown in a controlled environment, they are unexposed to external agents like pests and insects. Also, there is minimal or no contamination due to almost no external application of fertilizers or manures. Strategies concerning postharvest management and enhancing the shelf life of microgreens need to be particularly developed.

## 7 Strategies to overcome limitations of microgreen production

There are various strategies to encounter the limitations related to microgreen cultivation and production as presented through Figure 2 and discussed below.

### 7.1 Selection and validation of potential crops/genotypes for microgreen traits

Identifying a diverse collection of genotypes is very important to explore the most promising genotypes containing microgreens-related traits viz., high nutrition, shelf life, sensory attributes, acceptable taste, and yield. However, microgreens’ phytoactive compounds, antioxidant capacity, shelf life, and nutrient content depend on genotypes’ genetic makeup and environmental conditions. For instance, twenty diverse genotypes of lentil and mungbean were grown as microgreens in plain and high altitude two regions (Delhi and Leh Ladakh). The investigation for profiling of phytochemical, macro and micronutrients content along with antioxidant capacity was accomplished (Mishra et al., 2021). Based on phytochemical profiles, lentil genotype L830 and mungbean genotype MH810 were identified as superior to other genotypes for contents of total flavonoids, carotenoids, ascorbic acid, antioxidant parameters, and phenols. The difference in nutritional profiles of identical genotypes under different environmental conditions was observed, probably due to variable gene expression. However, the genes and pathways governing the variation in response to different environmental conditions are yet to be elucidated. This study has provided new insights into the potential application of microgreens in harbouring the nutritional security of inhabitants of harsh environments such as the high altitudes of Leh-Ladakh (Mishra et al., 2021). Some other legume microgreens viz., sainfoin, red clover, alfalfa, chickpea, lentils, maize, cowpea and mung bean were tested for phenolic, antioxidant activity, flavonoid, carotenoid, ascorbic acid, total chlorophyll, chlorophyll a, and chlorophyll b concentrations The highest total antioxidant activity (TAA: 4,789.373 mg TE g-1), total phenolic contents (TPC: 791.770 mg GAE 100–1 g-1) in red clover and highest total flavonoid content (672.177 mg QE 100 g-1) in maize were estimated (Altuner et al., 2022). Thus, it was concluded that total phenolic contents (TPC) was considerable good in red clover (46%) and maize (73%) as compared to cowpea and other studied legumes (Altuner et al., 2022). Although, biochemical parameters have not been correlated in legumes but pigment parameters were positively associated in legumes and cereals (Altuner, 2021). Further, positive correlation was found for total antioxidant activity, total phenolic content and total ascorbic acid in cereals (Altuner, 2021).

Variation in phytochemical and antioxidant profile and macronutrient composition of red and green butterhead lettuce cultivars (*Lactuca sativa* L. var. *Capitata*–green and red Salanova) has been reported with varying stages of development at harvest (El-Nakhel et al., 2020). Though microgreens of both cultivars were rich in calcium and magnesium, the red Salanova microgreens were concluded to be highly nutrient enriched. The nutritional profiles of Chicory and lettuce microgreens of the Asteraceae family and two genotypes of *Brassica* (broccoli) were compared. However, the *Brassica* microgreens were the richest source of phenols and vitamin E; the Asteraceae microgreens were rich in carotenoids and alpha-tocopherol (Paradiso et al., 2018).

These aforesaid studies suggest that microgreens with desirable nutritional contents can be obtained by exploring and manipulating the available genetic diversity.

### 7.2 Good agricultural practices (GAP) and optimized storage conditions for pre/post-harvest management to prolong the shelf life/quality of microgreens

Microgreens are preferred to be consumed afresh, either wholesome or as garnishes or seasonings and its cultivation at home can be an excellent practical approach concerning price and sustainability that also provides fresh life and functional food on the table for growing kids and families. Maintaining food safety standards with minimal or no microbial contamination that can cause potential health hazards without compromising the sensory qualities is of utmost importance.

Microbial contamination can cause spoilage of food and products, rendering them unfit for sale and consumption. This requires adopting hygienic and healthy practices throughout the cycle, beginning from cultivation until reaching the end user. Traditional methods like chilling, freezing, pasteurization and antimicrobial compounds (chemical or biological) compromise the sensory attributes (Tropea et al., 2021). Research is now focused on improving the quality and safety of food while maintaining its nutritional and organoleptic properties. One such technology is nisin-containing nano-carriers that can be applied safely as antimicrobial agents on food products (Bahrami et al., 2019).

Microgreens have a high respiration rate at the time of harvest that affects their shelf-life and storage (Chandra et al., 2012). Hence, application of 10 mM calcium chloride to microgreens prior to harvest is effective in delaying senescence, enhancing the visual appearance, and diminishing the growth of microorganisms during storage in broccoli microgreens (Kou et al., 2014). Further, Lu et al. (2018) studied the effect of applications of CaCl_2_ as pre-harvest and UV-B as post-harvest on levels of Glucosinolates (GLS) and glucoerucin (GLE) for assessing and enhancing the storage quality of microgreens. It was found that the treatments with 10 mM CaCl_2_ followed by UV-B enhanced GLS levels andcontent of total aliphatic glucosinolates in microgreens was four times as compared to mature counterparts. These microgreens had increased biomass, calcium content, and activities of antioxidant enzymes superoxide dismutase and peroxidase. Overall shelf life, productivity, and post-harvest of microgreens were improved.

The shelf life of microgreens is a very important concern that varies from 10–15 days after harvesting, depending on the category of microgreens. Microgreens are potential plant-based food/diet full of nutrition, fibers, and antioxidants, which diminish the risk of cardiovascular disease and numerous types of cancer. The microgreens may be packed in polypropylene bags and stored at 5°C in a climate chamber or incubator for 10 days with controlled temperature and humidity. However, the 1°C storage temperature was optimum due to no chilling injury (Xiao et al., 2014). A combination of pre-harvest and post-harvest treatments, different packaging materials, and modified atmosphere packaging (MAP) regulates the shelf-life of fresh-cut microgreens and diverse sensorial characteristics. Moreover, macro-perforated packaging, including PET clamshell and LDPE self-seal bags, was also assessed for longer shelf life in radish and roselle microgreens (Ghoora and Srividya, 2020).

After harvesting, packets of microgreens should be kept at a 4–5°C and consumed within 8–10 days. Storage conditions and maintenance of shelf-life are very important to preserve the microgreens in good quality with stable nutrition. Several factors, viz., storage temperature, atmospheric composition, post-harvest light exposure, and packaging technologies, are associated with conserving fresh-cut microgreens. Further, during value-added product development, processing avenues (freezing, drying, waving, microwaving, frying, toasting, and boiling)are equally required to maintain the bio-availability of bioactive and phytochemical components of microgreens.

Different technologies and methods have been explored to maintain microgreens’ shelf life and postharvest quality for preparing ready-to-eat products through wash steps and foliage spray. Aloe vera gel-based pre-harvest spray treatment and postharvest dip coatings were tested in radish and roselle microgreens for extended shelf life due to regulation of stomata closure (Ghoora and Srividya, 2020). These procedures regulate lower physiological weight loss, respiration rate, electrolyte leakage, microbial counts, and good overall acceptability. Further, researchers concluded that aloe vera gel-based-coated microgreens exhibit minimum deteriorative postharvest changes and higher ascorbic acid content than the uncoated control. Preharvest 10 mmolL−1 CaCl2 spray without postharvest dip displayed good yield, visual quality, and extended storage life (Kou et al., 2015). The optimum quality and highest shelf life of buckwheat microgreens can be maintained and stored at 5°C through moderately high O_2_ (14.0–16.5 kPa) and low CO_2_ (1.0–1.5 kPa) content with the treatment of chlorinated wash to reduce microbial counts (Kou et al., 2013). A very interesting plant regulator is 1-methyl cyclopropane (1-MCP) which binds competitively to ethylene receptors and delays senescence resulting in active treatment to prolong the shelf life of fruits, vegetables, and edible flowers (Turner et al., 2020). However, washing treatment is equally important in maintaining prolonged shelf life and the least microbial load. Comparative analysis of treated (chlorine wash) and controlled (unwashed) Ruby radish microgreens determines that 100 ppm chlorine wash enhances the visual quality and reduces electrolyte leakage (Turner et al., 2020). In addition, 0.25%–0.50% citric acid wash followed by 50% ethanol spray and 0.25% ascorbic acid is also effective in augmenting quality score. Further, to enhance the shelf life, a potential application of “nano packaging” technology concerning microgreens can also be explored for effective postharvest management. Thus, in coherence with the farm-to-fork tradition, good agricultural practices and handling practices are crucial.

### 7.3 Fortification through agronomic approaches, nano-technology and seed priming for enhancing preferred qualities of microgreens

Microgreens are considered potential nutrient sources that can help overcome the deficiency of many nutrients which are not met up with the seeds or mature parts of the plant. Effective fortification strategies for producing microgreens with desired nutritional traits and shelf life can be effective tools. Insufficient availability of iron and zinc in the human diet has posed a risk of malnutrition in young children and women. To address the deficiency of iron and zinc micronutrients, microgreen produce fortification can serve as an effective but short-term approach. Fortification can be done through several approaches such as agronomic practices, application of nanotechnology, *etc.*

Agronomic practices are cheap and simple but non-heritable and must be done with great care due to the application method, kind and environmental considerations. This strategy emphasizes improved nutrient accessibility to plants, efficient usage of nutrients, plant mobility, and increased microbial activity. Microbes like *Bacillus*, *Rhizobium*, *Azotobacte*r, *Actinomycete,* and some fungal strains, i.e., *Pseudomonas indica*, are used to increase nutrient availability and their uptake. Mineral nutrients show great potential for fortification when applied to the soil and the leaves. The most popular fertilizer is based on nitrogen, phosphorus, and potassium (NPK), which is vital for the health of both plants and mankind. Crops also require other micro-minerals such as iodine, zinc, copper, iron, nickel, molybdenum, manganese, *etc.*

In a recent study, the fortification of Brassicaceae microgreens was attempted for iron and zinc enrichment (Di Gioia et al., 2019). It involved growing red cabbage, red mustard, and arugula microgreens in nutrient solution supplemented with sulfate salts of iron and zinc at 0, 10, 20, and 40 mg L^−1^ and 0, 5, 10, and 20 mg L^−1^ concentrations, respectively. Further, investigations on the growth, yield, and mineral composition of these microgreens grown in these media composition exhibited accumulation of both iron and zinc minerals in microgreens of all three Brassicaceae members in a genotype-specific manner. Thus, this study also indicated that soil-less cultivation systems could be exploited for the production of fortification of microgreens by altering the composition of the nutrient medium. Pannico et al. (2020) have fortified selenium in green basil, purple basil, coriander, and tatsoi microgreens through a modified quarter-strength Hoagland nutrient solution with sodium selenite compound.

Nanofortification is the approach to fortify the plants or microgreens through nanoparticle application of some essential nutrients (Cu, Se, Fe, and Zn) in the form of liquid treatment as foliar and nano-fertilizers in soil or water medium (El-Ramady et al., 2021). Nanoparticles (NPs) are small materials ranging from 1 to 100 nm in size or dimension (Laurent et al., 2008), with a large surface area that allows its application in diverse fields, including fortification in plant systems. Their size and large surface area: volume ratio also contributes to their physical and chemical properties. Due to their size, the optical properties of these particles impart unique characteristic colours. Their size, property, and shape are categorized into different groups: metal NPs, ceramic NPs, polymeric NPs, and fullerenes. They find applications in several fields, including environmental engineering, biotechnology, textiles, food processing/packaging, cosmetics, plant sciences, and agriculture. Several methods are employed for the synthesis and detection of nanoparticles. The techniques used for synthesis include chemical synthesis, thermal decomposition, photo-reduction, and green synthesis. Further, characterization of synthesized NPs may be performed using UV-Vis spectroscopy, X-ray diffraction assay, Scanning electron microscopy (SEM), transmission electron microscopy (TEM), and energy dispersive analysis. The conventional chemical methods of NP synthesis are costly, and toxic chemicals used for synthesis also pose a hazard to the environment. These pave the way for the need to synthesize NPs from biological methods using plants, microorganisms, and enzymes. These methods are not only cost-effective and rapid but are also environment-friendly and safe. Several metal NPs have been synthesized using the green synthesis approach, like silver NPs, gold NPs, copper, zinc oxide, and iron oxide (Bibi et al., 2022; Eltaweil et al., 2022; Nguyen et al., 2022). These may be utilized for seed priming to improve germination, seedling growth, and nutrition. Several plant parts like roots, leaves, flowers, fruits, and stems have been used to synthesize NPs *via* green methods. For example, zinc oxide nanoparticles are stable oxides of metal, eco-friendly, and have no harmful effects on humans and animals. These are most interesting to researchers due to their magnetic, optical, thermal, and chemical properties. ZnO NPs also exhibit adsorption ability which increases the catalytic efficiency. Nanoparticles have been applied extensively in agriculture research and may be used as nano-fertilizers to promote plant growth.

Seed priming is an innovative and user-friendly approach to fortify seeds by treating with an appropriate number of desirable nanoparticles. Sundaria et al. (2019) investigated the effect of iron oxide nanoparticles (25–600 ppm) on wheat genotypes (WL711 and IITR2). They observed increased germination percentage, shoot length, growth parameters, and accumulation of grain iron in WL711 and IITR2 at 200ppm and 400ppm, respectively. In addition, cold plasma (CP) treatment is a pollution-free way to improve seed germination, water use efficiency, nutrient uptake, photo- and thermo-dormancy, and plant yield (Mahanta et al., 2022). Moreover, CP treatment would be a potential approach to improve microgreens performance as it plays a critical role in numerous physiological, biological, and developmental processes in plants that improve seed performance, bacterial load on seeds, altering seed coat structures, enhance seedlings growth and its association with machine learning is a sustainable approach for seed priming (Shelar et al., 2022).

### 7.4 OMICS and breeding approaches for microgreen biofortification

Microgreens contain various favourable attributes like a pleasing palette of colours, quality, textures, and flavours (Aroma volatiles associated with flavour) but limit their commercial use due to short shelf life. NASA scientists have also explored microgreens in space due to dynamic properties like the availability of oxygen generation, nitrogen, essential nutrients, and photoactive compounds to enhance the morale of astronauts during stretched stays away from Earth (Kyriacou et al., 2017). Several breeding approaches and multiOmics have been explored to augment the shelf life, developmental rate and nutrient content of vegetables and fruits (Kalia and Singh, 2018; Mathiazhagan et al., 2021; Reda et al., 2021; Valdes et al., 2021; AieseCigliano et al., 2022; Chakravorty et al., 2022; Dutta et al., 2022; Kumari et al., 2022; Parmar et al., 2022; Sahoo et al., 2022; Sharma et al., 2022). However, it has been observed in tomatoes that the dominance component was higher than the additive component for shelf life (Pavan and Gangaprasad, 2022). To maintain the shelf life with natural colour and flavour, an antisense gene was introduced in tomatoes by a Californian company Calgene in the 1980s and developed improved shelf-life tomatoes, i.e., popularly known as FlavrSavr tomatoes (Bruening and Lyons, 2000). In addition to that natural variant of cucumber fruit (DC-48:high shelf life) was also explored through qRT-PCR for fresh green colour and shelf life and found that Expansin (EXP), Polygalacturunase (PG), and xyloglucan endotransglucosylase linked with cell wall degradation process and regulates to maintain the fruit firmness (Pradeepkumara et al., 2022). Several such efforts are underway in several vegetable and fruit crops.

The term “biofortification” refers to the process of enhancing the nutritional value of a plant’s edible parts. It provides a long-term and sustainable alternative for supplying people with micronutrient-rich crops (Garg et al., 2018). Biofortification offers an effective, economically feasible, and sustainable means of enhancing nutritional content in crops that contribute to staple diets. This strategy typically involves interventions for improving the nutritional content, including vitamins, essential amino acids, minerals, and fatty acids, while simultaneously reducing anti-nutritive factors that hinder the bioavailability of nutrients in crop plants (Garcia-Casal et al., 2017). It has the potential to overcome malnutrition prevalent as ‘hidden hunger. In the current climate change scenario, where crop yield and nutritional quality are adversely affected, biofortification can be a successful and game-changing strategy for overcoming nutrient deficiencies, especially in developing nations. Biofortification can be done by understanding the genes, pathways and regulatory networks responsible for absorbing and transporting nutrients and adopting genetic means that involve using the natural germplasm during conventional breeding, genetic engineering (through genetic engineering or production of transgenics) and other OMICS approaches. The significance of multiomics, nutriomics and foodomics have been explained for development of desirable genotypes with potential microgreen related traits (shelf life and nutrient content) those will be also helpful for improvement of breeding cycles (Kalia and Singh, 2018; Mathiazhagan et al., 2021; Reda et al., 2021; Valdes et al., 2021; AieseCigliano et al., 2022; Bansal et al., 2022a; Chakravorty et al., 2022; Dutta et al., 2022; Kumari et al., 2022; Parmar et al., 2022; Sahoo et al., 2022; Sharma et al., 2022).

Conventional breeding depends on the genetic diversity of the gene pool for the trait of interest (TOI). The desired genes are pyramided using traditional crossing approaches, and then segregation populations are thoroughly screened. Biofortification *via* genetic means offers a cost-effective and relatively efficient strategy with the pre-requisite of the availability of inbred lines with high nutrient content in several crops. For millions of under privileged rural residents, sorghum is one of the most essential basic foods. It can flourish in challenging conditions. Moreover, HT12 protein increased the lysine content in sorghum (Zhao et al., 2003; Lipkie et al., 2013). The fact that sorghum is less easily digested than other main staple crops is one of the problems with eating it. Its kafirin seed storage protein is immune to protease digestion. The RNA silencing of kafirin increases the digestibility index of sorghum in combination with the suppression of kafirin-1, kafirin-2, and kafirin A1 genes (Grootboom et al., 2014; Elkonin et al., 2016). Accordingly, in soybean the globally preferred crop due to its vegetable oil and high-quality protein, the expression of the bacterial PSY gene (beta-carotene) increases the level of provitamin-A, oleic acid, and other protein contents of seed (Schmidt et al., 2015). The fruit color and freshness in strawberry were regulated by specific anthocyanin, anthocyanin related transcription factors and biosynthesis-associated gene expression (Lee et al., 2022). Thus, utilization of such nutrition-enriched developed varieties may be explored for microgreens production.

New breeding approaches, including transgenic breeding, RNA interference (RNAi), and genome editing etc. are crucial for the biofortification of crops because they provide new opportunities for developing unique genetic varieties and are being discussed under following sub heads.

### 7.5 Genomics and transcriptomics for microgreen traits

A number of DNA-based molecular markers viz; SSR (Simple sequence repeats),microRNA-based SSR, AFLP (Amplified fragment length polymorphism), SNP (Single Nucleotide Polymorphisms), etc. are available for diversity analysis and identification of most potential genotypes for desirable traits in different plants (Gupta et al., 2013; Maurya et al., 2015; Liu et al., 2019; Gupta et al., 2020a; Pradeepkumara et al., 2022) that would be useful to detect most potential genotypes based on phylogeny and genetics studies for particular genotype-specific microgreens. Nutritional profiling of the most diverse genotypes (range: 300–1,000 genotypes) for microgreen-related traits may be performed to detect major phytonutrient components. Evaluation of the most promising genotypes of potential plants (legumes, cereals, herbs, and vegetables) would be helpful for microgreen production based on performance for microgreen-related traits (aroma, tender texture, vivid colour, flavour, and rapid production)at different locations (high/low altitude). Genome-wide association mapping and quantitative trait loci (QTL) mapping will be helpful in detecting potential genotypes and for candidate gene identification related to microgreen related desirable traitsformolecular breeding programs.

Specific QTLs may be identified for desirable microgreens related traits (shelf life and nutrients content: Fe, Zn) in particular crop on the basis of contrasting parents and corresponding data of Genomics. The Quantitative Trait Loci have been identified for phytoactive compounds, iron, zinc and shelf-life related microgreen traits incabbage (Wu et al., 2008), broccoli (Gardner et al., 2016), wheat (Krishnappa et al., 2022), lettuce (Hayashi et al., 2012), melon (Dai et al., 2022)and chickpea (Mahto et al., 2022).

Accordingly, transcriptomics based specific mRNA expression quantitative trait loci (eQTLs)and splicing quantitative trait loci (sQTL) may also be identified for desirable microgreens related traits (shelf life, Fe, Zn) in particular crop on the basis of contrasting parents, standard population size and corresponding nutrition data (Agarwal et al., 2014; Zhu et al., 2022) as identified for several complex traits (Khokhar et al., 2019; Qi et al., 2022). Based on the desirable trait evaluation, a set of candidate genotypes may be selected for transcriptomics study to identify the differentially expressed genes (DEGs) for nutrition and related traits (Figure 2). The candidate genotypes should differ for traits, including bioactive compounds, phytochemicals, antioxidant capacities, mineral composition, yield, and biomass-related traits. The transcriptomic analysis will help to identify the differentially expressed transcripts, biological processes, and molecular pathways for all the contrasting traits, including nutritional and shelf-life-related traits. Once identified, the differential transcripts/genes will be converted into user-friendly markersto be utilized through breeding approaches for future microgreens production.

### 7.6 Proteomics and metabolomics for microgreen traits

Proteomics and metabolomics have been explored in several microgreens viz; broccoli (Sun et al., 2015), brassica (Castellaneta et al., 2022) and other leafy vegetables also (Sahoo et al., 2022). Novel datasets for microgreens may be generated through research activities, and prospects of encroachment in OMICS approaches. The improvements will consequence through the combined relationship of proteomics and metabolomics for nutritionally rich microgreen development. Protein specific quantitative trait loci (pQTLs), metabolic quantitative trait loci (mQTLs) and micronutrient quantitative trait loci (nutriQTL) play dynamic role in Physiological processes and molecular pathways that may be further identified for desirable microgreens related traits (shelf life, Fe, Zn) as investigated earlier (Engelken et al., 2016; Zhou et al., 2021). Furthermore, proteomics and metabolomics approaches will be a major advancement for microgreen improvement in terms of proteins, metabolites, and bioactive compounds. They will be helpful from plant breeding to OMICS-assisted plant molecular breeding (Langridge and Fleury, 2011).

Variations in the quality and quantity of microgreen proteins and metabolites can be investigated through analysis of proteome composition and changes to developmental stages, including stress-response mechanisms for the enhancement of proteome coverage data and further improvement of protein quality and shelf-life. The establishment of novel approaches related to proteomic and metabolites pipelines would be useful for data analysis associated with different kinds of growth and stress conditions for microgreen-related traits as already explored in several crops through proteome mapping, comparative analysis of proteomics, post-translational modifications, and protein-protein interaction networks, 2D gel electrophoresis coupled with MALDI-TOF (Vanderschuren et al., 2013; Katam et al., 2015). These approaches would be helpful for the purposeful annotation of desirable proteins that participate in metabolism (nitrogen, amino acid, carbon and energy, and Reactive Oxygen Species), stress response, secondary metabolism, and signal transduction (Gupta et al., 2019) that will be further regulated and improve nutrition quality and extended shelf-life.

Good accuracy, speed improvements, sensitivity perfections in mass spectrometry (MS) applications, and software tool improvements have all benefitted high-throughput protein quantification that will be further useful for comparative analysis of proteomics profile in association with differential expression analysis as explored related to stress responses in legume crops (Pandey et al., 2008; Abdallah et al., 2012; Hu et al., 2015).

Metabolite profiling provides the appropriate data and depth information on metabolic networks responsible for a diverse range of desirable phenotypic traits and undesirable traits that can be regulated through plant metabolic engineering (Fernie and Schauer, 2009). The literature highlights two important nuclear magnetic resonance (NMR) and mass spectrometry (MS)-based metabolomics profiling techniques. It was usually necessary to combine several analytical methods to extract a wider variety of multiple plant metabolites from a single MS (Arbona et al., 2013). Additional methods include Fourier Transform Infrared spectroscopy and MS (FIA/MS), and flow injection-based analysis.

Integrating metabolomics, transcriptomics, bioinformatics platforms, and phenomics to evaluate genetically diverse individuals and improve gene identification accuracy enables the detection of unique metabolic QTLs and candidate genes for the targeted trait that will be cooperative for microgreens improvements. Moreover, a combination of metabolomics screening and genomic-assisted selection strategy has been identified to increase yields, reducing the time spent discovering novel traits and allelic mutations (Fernie and Schauer, 2009).

### 7.7 Pan genomics for microgreen traits

A species’ pan-genome refers to all of its genes collectively. Due to the variety in genomic sequences, it has been determined that a single organism cannot have all of a species’ genes. Completeness (i.e., the presence of all functioning genes), stability (i.e., the presence of distinctive catechistic properties), comprehensibility (i.e., the presence of all genomic data for all species or individuals), and effectiveness are the desired characteristics of an ideal pan-genome (i.e., organized data structure). Recently, a 592.58 Mb chickpea pangenome with 29,870 genes was created (Varshney et al., 2021). In order to create the pan-genome, 3,366 accessions totalling 3,171 farmed and 195 wild ones were used in whole genome sequencing. This comprehensive genome analysis provided important details on the genomic regions frequently chosen during domestication, the best haplotypes, and the locations of harmful allele targets. The newly discovered genes that encode reactions to oxidative stress, stimuli, heat shock proteins, cellular (acidic pH) and cold responses may help to modify microgreen cultivation.

### 7.8 Transgenic approaches for microgreen traits

In order to introduce tolerance or resistance to diverse abiotic and biotic problems, genetic manipulation has been extensively used to identify and transfer resistant gene(s) from a variety of resources to desirable plants for a targeted trait. Today, different genes are used in plants, and transgenic plants have been created through *Agrobacterium*-mediated transformation (Sharma et al., 2006), electroporation of intact axillary buds (Chowrira et al., 1996), particle gun bombardment (Indurker et al., 2007). *Agrobacterium*-mediated explant transformation is the most frequently employed technique to create transgenic pulse crops by inserting transgenes from diverse sources to produce transgenic plants.

Various transgenic plants have been developed for several desired traits. Further, several genes have also been identified for insect pest (protease inhibitor genes, α-amylase inhibitor genes, lectin genes, Cry genes from *Bacillus thuringiensis*, chitinase gene) and disease resistance for example fungal (antifungal protein genes, stilbene synthase gene), viral (coat protein genes of viruses) and bacterial (T4 lysozyme gene) (Dita et al., 2006; Eapen, 2008). The impact of endogenous genes could be regulated by modifying biological processes and metabolic pathways to boost carotenoids and flavanoids using various abiotic stimuli such as drought, salinity, mineral toxins, cold, temperature and RNA interference technologies (Eapen, 2008). Interestingly, desirable multi-trait transgenic plant can be developed through *in vitro* gene stacking system: GuanNan Stacking (Qin et al., 2022). However, transgenic rice have been developed by construction of binary vector and insertion of five desirable foreign genes that would be helpful for regulation of metabolic engineering and trait improvements through breeding and multiomics approaches in future (Qin et al., 2022). Moreover, improvement of chickpea and pigeonpea have been explored through transgenic and molecular approaches (Arya and Mishra 2022).

### 7.9 Genome editing for microgreen traits

In plant genome editing, sequence-specific nucleases modify specific genes in the selected crop to construct transgene free plants. Moreover, different sequence-specific nucleases including ZFNs, TALENs, and the CRISPR-Cas9 systems have been explored to alter the genome of the targeted plant, fruits and vegetables (Upadhyaya et al., 2009; Mathiazhagan et al., 2021; Chakravorty et al., 2022; Kumari et al., 2022). CRISPR genome editing uses RNA-guided DNA endonucleases (Cas9/13), but these complexes form at the specific target site to execute targeted gene editing (Ducreux et al., 2004; Dancs et al., 2008). Enhancing shelf life and plant storage features by genome editing would be a valuable venture (Kuzmina, 2020). Although, biofortification for cytokinin using gene editing for improving nutrition in chickpeas has recently been projected (Mahto et al., 2022).

### 7.10 Sequencing-based approaches for microgreen traits

With the advances in the NGS based technologies, trait mapping has become an easy job to do. Not only are these technologies time saving but also reduces the cost at basal levels. The genetic mapping is based on recombination (the exchange of DNA sequence between sister chromatids during meiosis) and the centimorgan (cM) distance measured between the markers by representing approximately 1% of the recombination frequency, while the physical map is based on the alignment of the DNA sequences with distance between markers measured in base pairs. However, the high-resolution physical maps serve as the scaffold for genome sequence assembly to identify the most accurate distance between the markers and the genes linked in addition to exploring of the potential candidate gene(s) linked to desired traits. The trait mapping through sequencing approaches may be categorized into two classes i) Sequencing of complete populations for trait mapping and ii) Sequencing of pooled samples for trait mapping (Singh et al., 2022). Researchers have great interest in its genomic properties, which provides a valuable marker for crop improvement. Wu et al. (2020) identified multiple high-quality SNPs that would serve as an important resource for the mungbean’s nutritional improvement and cultivation. Due to the advancement in sequencing techniques, Dasgupta et al. (2021) conducted the bulk-RNASeq-based gene expression analysis across mungbean genotypes to identify disease-resistance genes. Bhardwaj et al. (2015) did an RNAseq-based analysis to identify drought stress-regulated genes in *Brassica juncea*. Guo et al. (2021) emphasized the genomic-based structural variant that indicated the diversification of different morphotypes of *Brassica oleracea*. These approaches are equally applicable and may be explored for improving microgreen traits (Mishra et al., 2021).

### 7.12 Epigenomics for microgreen traits

The increasing world population and changing climate increase the demand for greater crop productivity. The selection of appropriate genetic techniques and desirable heritable DNA sequences have led to notable genetic advancements in many crop species. Specific methylation quantitative trait loci (meQTLs) may be identified for desirable microgreens related traits (shelf life, Fe, Zn) (Kumar et al., 2016; Kumar et al., 2022b). Further, correlation between transcriptome (eQTL) and methylome (meQTLs) have been established for genetic regulation of complex traits (Oliva et al., 2022). In addition, a better comprehension of and capacity to choose advantageous epigenomic modifications is suggested to incorporate a more effective and comprehensive approach to crop improvement (AieseCigliano et al., 2022; Chandana et al., 2022). This is because many plant stress responses are governed by epigenomic processes, notably through cell-autonomous epigenetic switching. This makes it possible to register and remember random genetic signals. According to a report, the memory-directed alteration may result in an increased ability to resist stress in the future (Berr et al., 2011).

The mechanisms governing plant-stress interactions and conditions are revealed by studying the roles of epigenetics causing stressors, such as histone changes and DNA methylations (Chinnusamy and Zhu, 2009). Numerous heritable modifications originating from mitotic and meiotic divisions (variations in the heredity of epigenetic markers) were observed during gene expression studies and are steadily transmitted from one generation to the next that were not encoded in the DNA sequence itself (Tsaftaris and Polidors, 2000; Berger et al., 2009; Chen et al., 2010).

Stout epigenetic alterations are mitotically transmitted through genomic imprinting, but transient epigenetic alterations are not heritable (Spillane et al., 2001). Until they are lost or removed, epigenetic alterations produced during meiosis are always transmissible from one generation to the next without the need for initial stimulation. The loss could be from a genetic mutation, unintentional (for unknown reasons), or result from environmental factors. These are distinct from the ones that brought about the original epigenetic changes. In plants, heterosis is exhibited in hybrids for high biomass (Gupta et al., 2020b), a straightforward epigenetic assumption.

### 7.13 Genomics to artificial intelligence for microgreen traits

Machine learning (ML) and Deep Learning (DL) is the component of artificial intelligence (AI) that includes mathematical models of data to improve plant performance based on statistics, predictive modeling, and data analysis. It may be considered artificial intelligence based on plant breeding or crop improvement. Precision agriculture and crop improvement through artificial intelligence are new for plants and are untouched areas for microgreens. Prediction of shelf-life is also possible through exact measurement of plant characters (accurate interpretation of high-throughput phenotypic data) by good quality imaging techniques (greater than 7,000 high-resolution images of 300 GBytes) and proficient analysis of refined extracted data using artificial intelligence.

**FIGURE 1:**
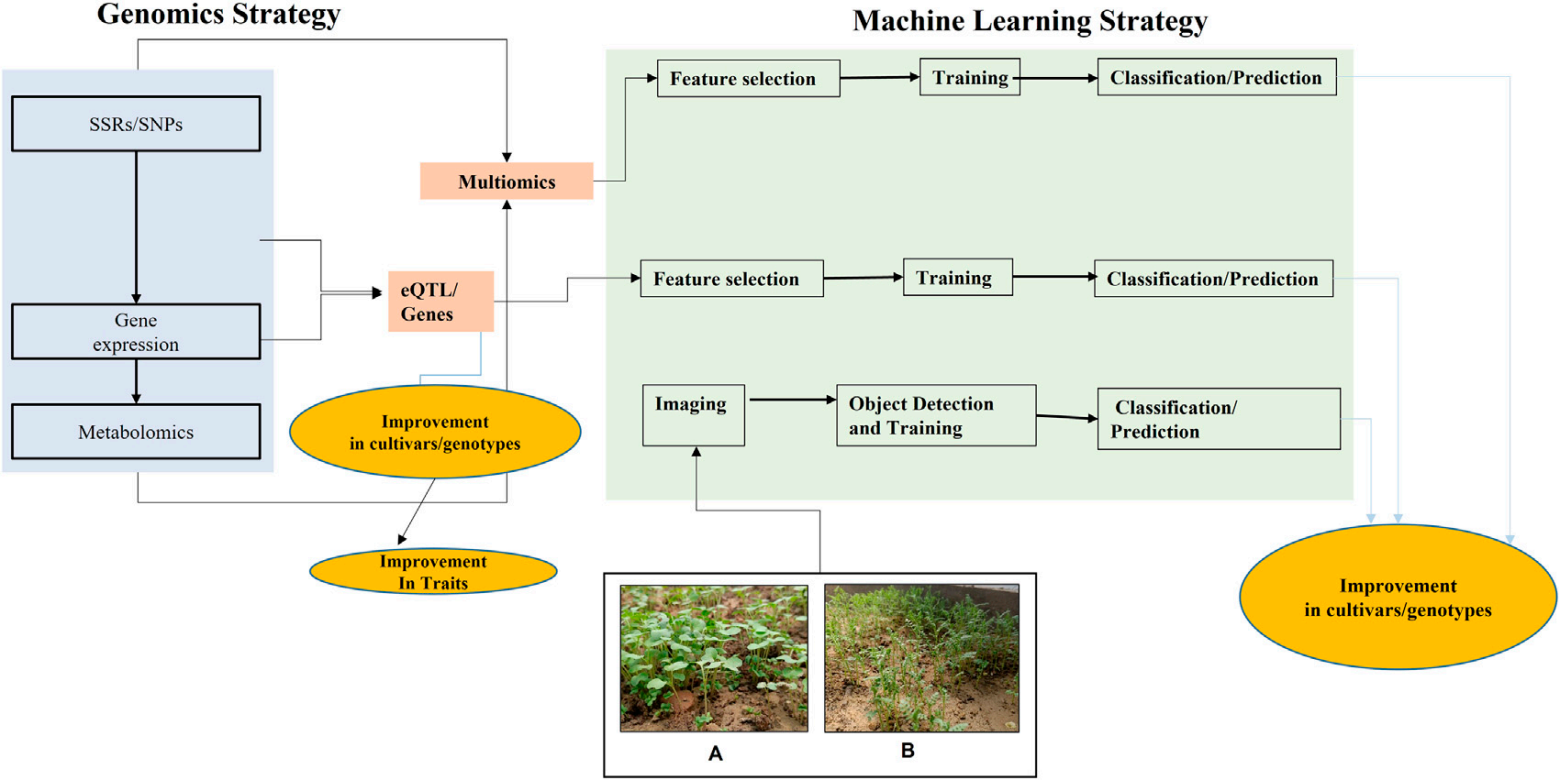
Systematic workflow of OMICS analysis and its association with machine learning for microgreens improvement and shelf-life prediction: Genomic data could be used to explore the markers in the form of SSRs, SNPs. Bulk-RNASeq and metabolomics data provides another layer of information that would enable Multi-OMICS analysis for cultivation improvement. Machine learning strategies that make the use of both “numeric” and “imaging” data would enable the development of prediction and classification models.

Microgreens-specific genomics and machine learning-based innovative approaches may be employed in other crop plants that have been proposed worldwide. Genomics approaches help to identify the “SNP” and “SSR '' markers and annotate the genes. Furthermore, a detailed analysis of these markers could help identify the nearest genes (QTLs), as shown in Figure 1. Another important genomic layer of information is “Bulk-RNASeq,” which can be implemented for accurate data analysis and prediction. ML aims at providing innovative approaches for prediction-based model development; Girma (2019) discussed the ML approach in mungbean to classify the raw quality of samples by analyzing digital imaging data; Jung et al. (2021) applied the deep learning algorithm to correctly identify the *Brassica napa* varieties, followed by a cross-validation approach. Another important microgreen vegetable crop is Broccoli (*Brassica oleracea* L. var. *italica*). The importance lies in the broccoli head portion, which helps to assess plant quality and different biotic and abiotic stress. Zhou et al. (2020) used the “Improved ResNet” to extract the broccoli pixels from the background data. In another study based on broccoli head estimation, Kusumam et al. (2017) applied the deep learning approach for 3D-vision-based detection. DL based semantic segmentation models have been applied in another microgreen plant cabbage for crop estimation (Jo et al., 2021). One of the studies by Wolanin et al. (2020) used the DL method for the time series data to estimate the wheat yield in the Indian wheat belt. AI has been applied to the barley seeds too. Various studies have been published where machine learning has been used for the identification of lentil-based rust disease identification (Singh et al., 2019). Applying machine learning models and techniques predicted the shelf life of Okra (Iorliam et al., 2021) and muskmelons (Albert-Weiss and Osman, 2022). Thus, active learning would be helpful to predict the shelf life of different types of microgreens through Support Vector Machine, Logistic Regression K-Nearest Neighbour algorithms,Naïve Bayes and Decision Tree.

### 7.14 Bioinformatics and molecular databases for microgreen traits

The plant research group requires efficient bioinformatics pipelines and a system to support efforts to analyse microgreen-related targeted plant genomes through functional genomics due to the rapid progress of publicly available databases from different kinds of tissues, development, environments, and stress treatment. The comprehensive model plant genomics, transcriptomics, and proteomics databases can be used to identify appropriate microgreen genotypes. The genome sequences of several plants (*Medicago truncatula*, *Glycine max*, and *Lotus japonicus*) and reference plant species (*Arabidopsis thaliana* and *Populus trichocarpa*) are already available to investigate gene function, biological processes, metabolic pathways, and genome evolution (Li et al., 2012). The available data bases viz., The Legume Information System (LIS; https://legumeinfo.org) and KnowPulse (https://knowpulse.usask.ca)are very informative and useful computational genomics platforms to evaluate molecular markers, diversity analysis, comparative genomics, gene annotation, novel transcription factors, sequence variants, phenotypic traits informationand to map SNPs, QTLs, long non-coding RNAs and to identify candidate genesfor selection of microgreen oriented suitable chickpea, faba bean, common bean, lentil, andfield pea germplasm (Doddamani et al., 2015; Verma et al., 2015; Dash et al., 2016; Gayaliet al., 2016; Sanderson et al., 2019; Lee et al., 2022).

## 8 Integrating various OMICS approaches for microgreen traits

Prospective OMICS approaches have been investigated in many plants to improve the desirable traits and elucidated earlier (AieseCigliano et al., 2022; Chakravorty et al., 2022; Dutta et al., 2022; Gupta, 2022; Gupta and Tyagi, 2022; Parmar et al., 2022; Sharma and Gupta, 2022; Singh et al., 2022). Re-sequencing activities of whole-genome employing genetic diversity, domestication patterns, evolutionary analysis, population structure, and linkage disequilibrium for chickpea improvement as a result of the technical advancements that upgraded chickpea (an orphan crop) to a potential geneticcrop (Varshney et al., 2019).

**FIGURE 2:**
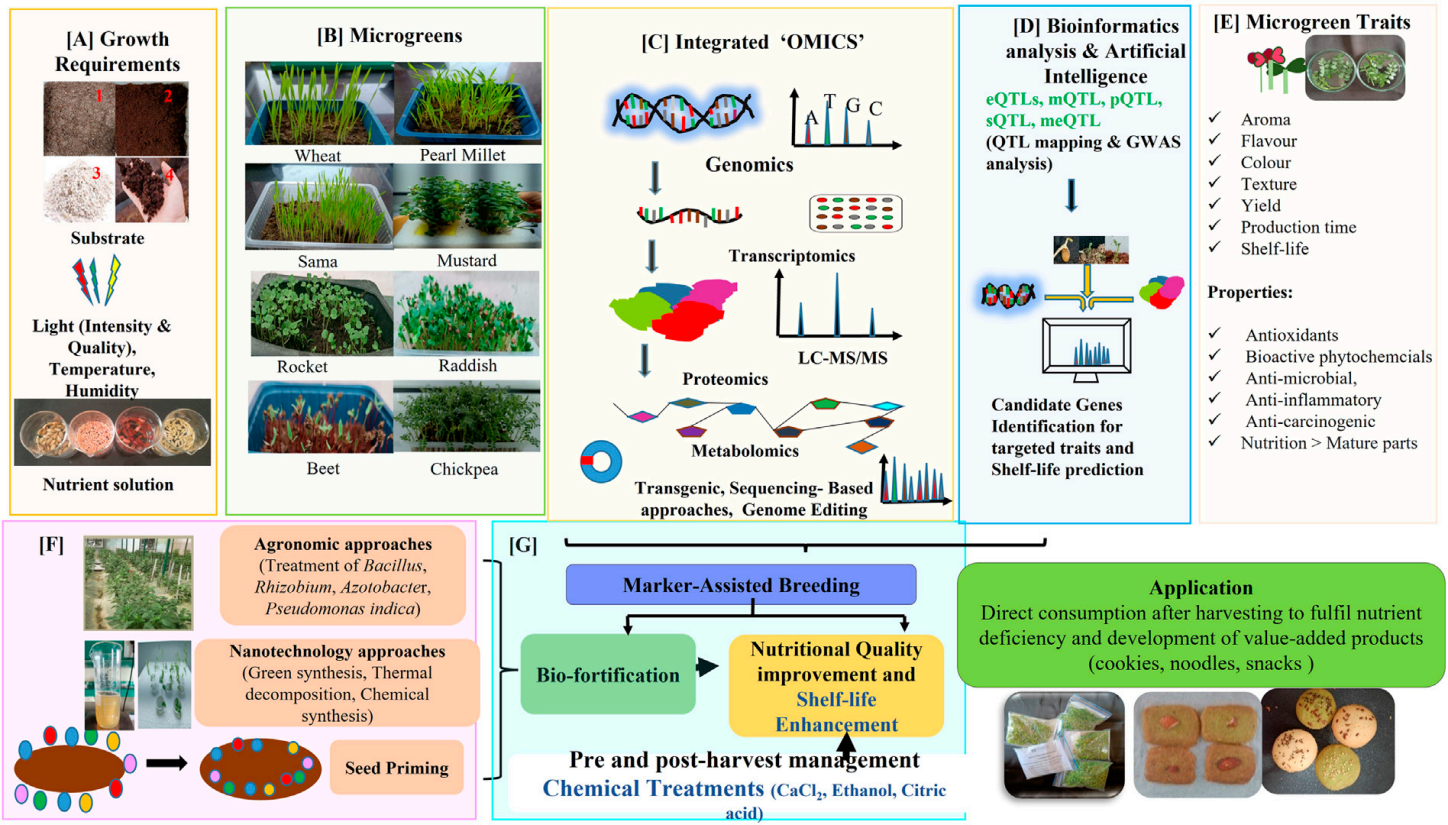
Summarizes prospects of microgreens as budding live functional food. **(A)** Growth conditions required for microgreens cultivation which includes a variety of substrates like vermiculite (1), cocopeat (2), perlite (3) and vermicompost (4), light (quality, intensity and duration), temperature, humidity and nutrient solution. **(B)** Microgreens of different plant species: Wheat, Pearl millet, Sama, Mustard, Rocket, Raddish, Beet and Chickpea. **(C)** Numerous “OMICS’ approaches such as Genomics, Transcriptomics, Proteomics, Metabolomics, Epigenomics, along with Transgenics, Gene editing and Sequencing based approaches can be integrated with bioinformatics tools and artificial intelligence **(D)** to tag Quantitative Trait Loci (eQTLs: mRNA expression Quantitative Trait Loci; meQTLs: Methylation Quantitative Trait Loci; PQTL: Protein Quantitative Trait Loci; sQTL: splicing Quantitative Trait Loci; mQTLs: Metabolic Quantitative Trait Loci) and candidate genes identification for microgreen related desirable traits **(E)** like nutrients, flavour, colour, early germination, yield and shelf life. The data generated from Integrated “OMICS’ approaches can be further utilized in molecular breeding to produce nutritionally rich varieties with improved shelf life through Marker-Assisted Breeding and producing biofortified microgreens with targeted micro/macro nutrients (like iron, zinc, magnesium, calcium) enrichments. **(F)** Biofortification of target microgreen is also possible by agronomic approaches (incubation with microorganisms like *Bacillus, Rhizobium, Azotobacter, Pseudomonas indica*), nanotechnology (nano-biofortification) and seed priming. Bioavailability of nutrients and minerals of microgreens can be stabilized through pre and post-harvest management strategy **(G)**. Improved microgreens with desired nutrients can be either consumed fresh as garnishes in soups, sandwiches, salads or processed to develop value-added products (like noodles, breads, drinks, cookies *etc.*) to overcome nutrients deficiency.

Recent genomics methods offer the potential to accelerate gene discovery, marker creation, molecular breeding, trait mapping, and productivity advances in microgreens, among other processes (Figure 2). Integration of precise phenotypic variation, low-frequency variants, and sequence information approach would be helpful for the selection of the most appropriate accessions with desirable key traits like biomass components, biotic and abiotic stress tolerance, and nutritional traits (Roorkiwal et al., 2020). A broad range of molecular markers (SSR, SNP, and DArT) have been discovered in chickpea that has been facilitated by NGS technology (Whole-genome re-sequencing, genotyping by sequencing, skim sequencing, RAD-Seq, and lower-depth sequencing). They can also be used for the development of chickpea microgreens (Kale et al., 2015; Varshney, 2016; Varshney et al., 2018).

There is a huge gap from genome to phenome in agricultural plants to identify the particular phenotype based on their DNA sequence information and genetics. Thus, it is crucial to integrate multi-OMICS information in one place from several branches of OMICs platforms, including phenomics, genomics transcriptomics, proteomics, epigenomics, and metabolomics. Using all the OMICS technology, the genotype-phenotype divide in any microgreens can be closed with precision phenotyping.

To learn new things about the potential genes and biological processes involved, analysis at genomics, transcriptomics, proteomics, epigenomics, and metabolomics levels can be done depending on the study’s goal. Moreover, it has been reported by Mannur et al. (2019) that *Fusarium* wilt resistance loci (foc 4) from WR 315 Annigeri 1 has been made available as “Super Annigeri one″ for commercial production in India using a genomics strategy. Thus, it is evident that various other studies have also proposed OMICS-based integration methods (Argelaguet et al., 2018; Bhardwaj and Steen, 2020; Mahto et al., 2022). Cai et al., 2021 discussed applicability of OMICS data (transcriptome and proteomics) for barley and identified the connection between sugar metabolism and wild barley. Li et al. (2019) used a single omics layer of information, i.e., transcriptome, and revealed the gene expression patterns of sulforaphane metabolism in Broccoli florets. For microgreens, this field is still lagging (Figure 1). There is a need to exploit the information from different resources to identify various biological factors that would help microgreen plants’ cultivation and breeding processes.

Candidate genes have been identified and incorporated in desirable plant through Genomics (QTL mapping), transcriptomics and transgenic approaches for microgreens related traits (shelf life and nutrient content: Fe, Zn) in different plant systems viz cabbage (Wu et al., 2008), broccoli (Gardner et al., 2016), wheat (Krishnappa et al., 2022), lettuce (Hayashi et al., 2012), melon (Dai et al., 2022) and chickpea (Mahto et al., 2022). Metabolomics and proteomics have been explored in several microgreens viz., broccoli (Sun et al., 2015), brassica (Castellaneta et al., 2022) and other leafy vegetables also (Sahoo et al., 2022). Thus, it is evident that we may identify and predict candidate genes associated with microgreens related traits (shelf life, desirable nutrients content, developmental rate and phytochemicals) may be incorporated in targeted crop varieties of fruits and vegetables utilizing the crop specific data bases of genomics, transcriptomics and metabolomics, marker-assisted selection, GWAS, bioinformatics, AI approaches utilizing the databases of specific crop transgenic CRISPR/Cas9 and gene editing approaches (Mathiazhagan et al., 2021; Valdes et al., 2021; Chakravorty et al., 2022; Parmar et al., 2022; Sahoo et al., 2022)**.** Thus combination of multiomics, foodomics in association with nutriomics would be fruitful for regulation of nutritional balance, health management and treatment of diseases (Bansal et al., 2022a).

## 9 Conclusion and future perspective

Optimization of light, substrate, and temperature would be helpful for good quality microgreen cultivation containing desirable aroma traits, tender texture, vivid colour, flavour, sensory attributes, and rapid production. Further evaluation of genotypes would be helpful for the selection of the most potential genotypes for the mass production of microgreens. Genomics and transcriptomics approaches may be explored for candidate gene identification for microgreens and important nutritional traits. Explored genes or associated SNPs may be developed and explored for user-friendly markers for marker-assisted selection and metabolic pathways with the integration of multi-OMICS approaches. Characterization of microgreens can be performed by combining the most standardized condition for the growth of microgreens with enhanced nutrient quality and bioavailability. Profiling of phytochemicals, nutrients, and minerals should be studied for good quality microgreens and biofortify further using traditional and novel biofortification approaches. For commercialization and popularization of the microgreen’s cultivation and harvest, shelf-life should be focused. The development of post-harvest technology for enhanced storability of microgreens is very important for synthesizing value-added, tasty, and nutritional products.

Due to the nutritional content inclusion of microgreens in the diet have several health benefits, as evident from the literature. However, microgreens’ growth, yield, and nutritional content can vary with the growing method (soil, compost, or hydroponic), intensity and quality of illumination, and composition of plant nutrient solution. Establishing hydroponics and vertical farming systems will enhance the cultivation of nutritionally rich microgreens with an easy harvest. The development of technologies to preserve microgreens is needed for a more extended period with minimal changes in their phytochemicals and nutrients. Novel value-added products (for example, drink, juices, cookies, noodles, *etc.*) may be developed with the integration of microgreens as one of the ingredients having wider acceptability and enhanced nutrition, especially for elderly persons, infants, young growing children, and sick persons due to excellent digestibility. Microgreens cultivation is a very easy and promising strategy to initiate at home or as a start-up for beginners/poor farmers in their respective localities, including people with malnutrition in India. The persons engaged in microgreens cultivation, production, and utilization may explore the food processing and packaging industry to enhance the market regarding agriculture and nutritionally rich products. Thus, microgreens will occupy a central place in the future food industry.\
